# CD133/*Prom1* marks proximal mouse oviduct epithelial progenitors and adult epithelial cells with a low generative capacity

**DOI:** 10.1242/bio.059963

**Published:** 2023-09-13

**Authors:** Matthew J. Ford, Keerthana Harwalkar, Hengameh Kazemdarvish, Nobuko Yamanaka, Yojiro Yamanaka

**Affiliations:** Goodman Cancer Institute, Department of Human Genetics, McGill University, Montreal QC H3A 1A3, Canada

**Keywords:** *Prom1*, CD133, Oviduct, Fallopian tube, Homeostasis, Stem cell, Progenitor, Epithelium, Multiciliated cell

## Abstract

The epithelium lining the oviduct or fallopian tube consists of multiciliated and secretory cells, which support fertilization and preimplantation development, however, its homeostasis remains poorly understood. CD133/*Prom1* expression has been used as a marker to identify adult stem cell populations in various organs and often associated with cancer cells that have stem-like properties. Using an antibody targeted to CD133 and a Cre recombinase-based lineage tracing strategy, we found that CD133/*Prom1* expression is not associated with a stem/progenitor population in the oviduct but marked predominantly multiciliated cells with a low generative capacity. Additionally, we have shown that CD133 is disparately localised along the oviduct during neonatal development, and that *Prom1* expressing secretory cells in the ampulla rapidly transitioned to multiciliated cells and progressively migrated to the ridge of epithelial folds.

## Teaser

**Summary:** A description of CD133/Prom1 expressing epithelial cells in adult and postnatal mice identifying differences between distal and proximal domains and no evidence of a resident stem cell.

## INTRODUCTION

*Prom1* (Prominin-1) is a transmembrane glycoprotein whose expression was first characterized on the surface of hematopoietic stem cells using the antigen CD133 (Yin et al., 1997; Miraglia et al., 1997). *Prom1*/CD133 is now recognized as an adult stem cell marker and has been used to isolate various tissue-specific stem cell populations across multiple organs (Uchida et al., 2000; Sagrinati et al., 2006; Richardson et al., 2004). CD133 has also been identified on the surface of cancer cells with stem cell like properties in many tumors including ovarian, liver, brain, prostate, colon, hepatocellular and lung cancer (Glumac and Lebeau, 2018). The specificity of *Prom1*/CD133 as an adult stem cell marker has however come into question due to conflicting reports of CD133 specification, potentially due to the use of different antibodies between studies, and the broader expression pattern seen in *Prom1* reporter mouse lines, indicating potential differences between CD133 antibody stainings and *Prom1* expression (Weigmann et al., 1997; Pfenninger et al., 2007; Florek et al., 2005; Shmelkov et al., 2008; Snippert et al., 2009; Zhu et al., 2009, 2016). In a lineage tracing study of *Prom1* expressing cells, Zhu and colleagues found variation in the generative and proliferative capacity of *Prom1* expressing cells between organs (Zhu et al., 2016). It was found that the *Prom1* expressing cells with a high generative capacity had a higher risk of tumor formation after an oncogenic insult, indicating that the stem cell characteristics of *Prom1* expressing cells varies between different organs and that locating those populations with a high generative capacity may identify highly susceptible cells to malignant transformation.

The oviduct epithelium is constituted of multiciliated and secretory cells, that aid the transport, function and survival of gametes and embryos (Li and Winuthayanon, 2017). It has been shown by lineage-tracing studies that secretory cells are proliferative and can differentiate into multiciliated cells, suggesting that secretory cells represent a bipotent progenitor (Ghosh, Syed, and Tanwar, 2017). Mounting evidence suggests that these cells are also the cell-of-origin in many cases of high-grade serous ovarian carcinoma (HGSOC), the most common and aggressive form of ovarian cancer (Kroeger and Drapkin, 2017). The presence of an adult stem cell, i.e., an undifferentiated cell whose progeny replenish dying cells, has not been characterized in the oviduct. Multiple studies have however eluded to the presence of a resident adult stem cell by the identification of label-retaining cells and organoid forming cells, enriched in the distal oviduct (Patterson and Pru, 2013; Wang et al., 2012; Xie et al., 2018; Paik et al., 2012). The expression of several common adult stem cell markers has also been reported, such as *Cd44*, *Prom1* and *Lgr5* (Almasry et al., 2018; Alwosaibai et al., 2017; Ng et al., 2013). However, there have been no *in vivo* characterizations of these populations that confirm their status as a progenitor or adult stem cell.

In this study, we investigated the stem cell properties of CD133/*Prom1*-expressing cells in the mouse oviduct epithelium using Cre-based lineage tracing techniques and CD133 antibody staining in adult and neonatal mice. We found that CD133/*Prom1* expression is limited to proximal epithelial progenitors and then becomes restricted to a sub population of oviduct epithelial cells, that are predominantly multiciliated, along the length of the oviduct. Our lineage tracing experiments revealed that *Prom1* expressing secretory cells in the ampulla rapidly differentiated to multiciliated cells and progressively moved from the base to the ridge of epithelial folds but remain restricted to the base of epithelial folds in the isthmus. Taken together, our results highlight the developmental and homeostatic differences between distal and proximal populations and provide no evidence of a resident adult stem cell population in the mouse oviduct epithelium.

## RESULTS

### CD133 is disparately localized along the mouse oviduct epithelium on the apical surfaces of secretory and multiciliated cells

**Fig. 1.**
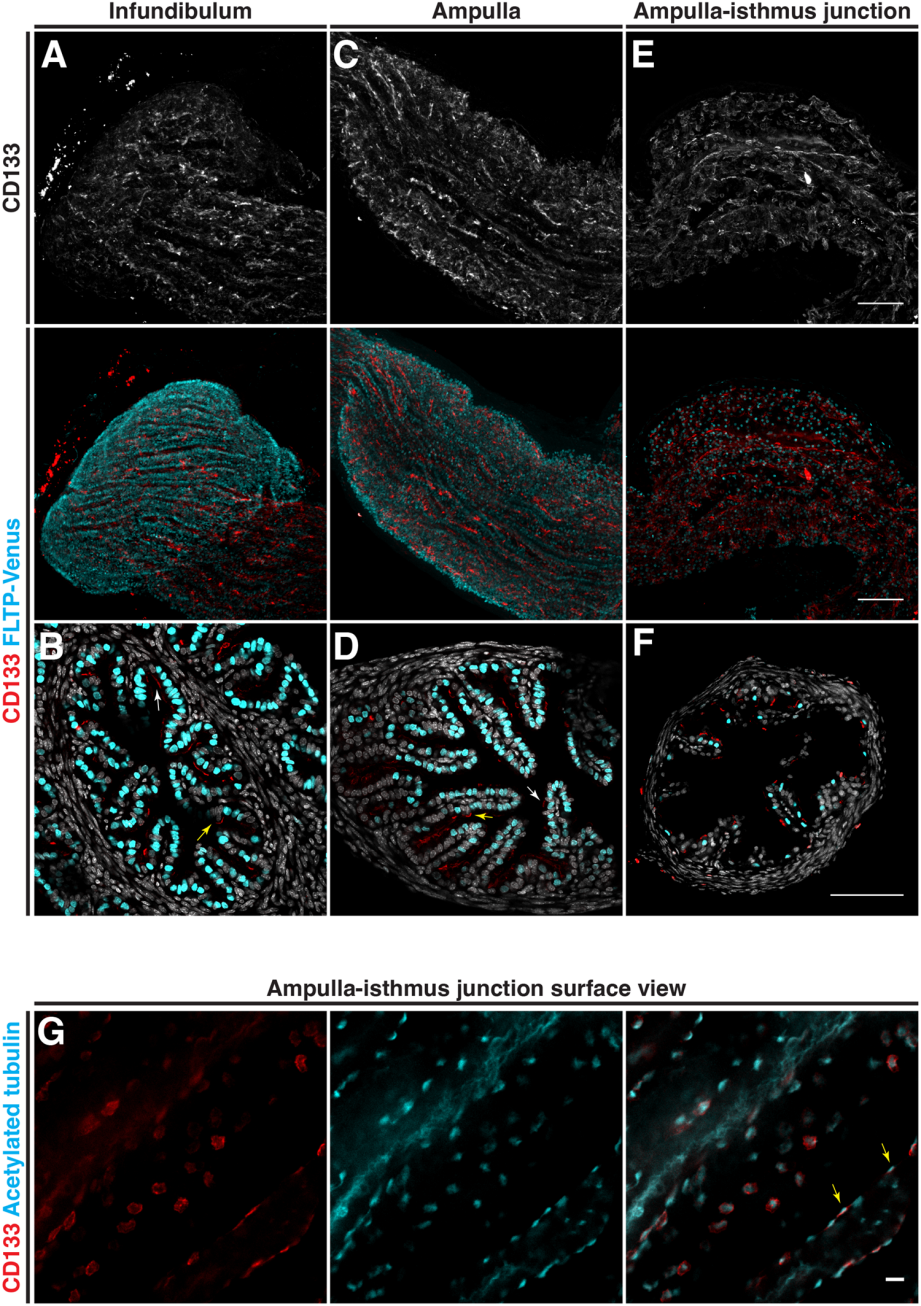
**Distribution of CD133 in the distal adult mouse oviduct.** The distribution patten of CD133 analyzed by immunostaining in *FLTP-H2B-Venus* mice. (A) Whole-mount Z-projection of the infundibulum showing extensive staining along epithelial folds with multiciliated cells labeled with FLTP-Venus. (B) A transverse section of the infundibulum showing CD133 associated with FLTP-Venus labeled epithelial cells. (C) Whole-mount Z-projection of the ampulla showing extensive staining along epithelial folds. (D) A transverse section of the ampulla showing CD133 associated with FLTP-Venus labeled (white arrow) and unlabeled (yellow arrow) epithelial cells. (E) Whole-mount Z-projection of the ampulla-isthmus junction, showing discrete staining of CD133 associated with multiciliated cells labeled with FLTP-Venus. (F) A transverse section of the ampulla-isthmus junction showing CD133 associated with FLTP-Venus labeled (white arrow) and unlabeled (yellow arrow) epithelial cells. (G) Whole-mount imaging of the ampulla-isthmus junction opened and imaged from the surface. CD133 is localized to the apical surface of multiciliated epithelial cells basal to cilia labeled with acetylated tubulin (yellow arrow). Scale bars: A-F, 100 µm; G, 10 µm.

**Fig. 2.**
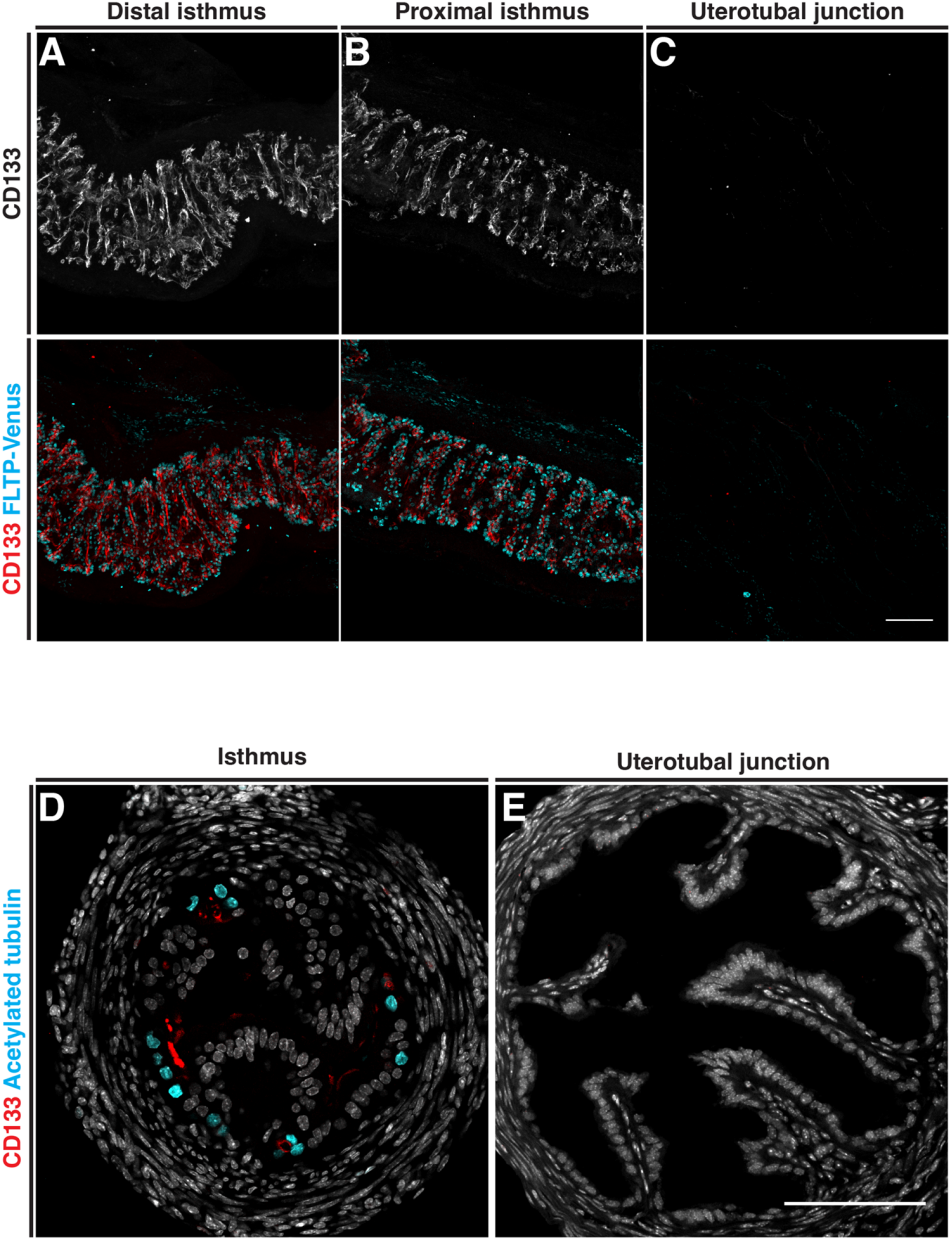
**Distribution of CD133 in the proximal adult mouse oviduct.** The distribution patten of CD133 analyzed by immunostaining in *FLTP-H2B-Venus* mice. (A,B) Whole-mount Z-projection of the distal and proximal isthmus showing CD133 staining along transverse epithelial folds associated with multiciliated cells labeled with FLTP-Venus. (C) Whole-mount Z-projection of the uterotubal junction show no CD133 staining or FLTP-Venus cells. (D) A transverse section of the isthmus showing CD133 on the apical surface of FLTP-Venus multiciliated epithelial cells located at the base of epithelial folds. (E) A transverse section of the uterotubal junction confirming the absence of CD133 and FLTP-Venus cells. Scale bars: 100 µm.

To determine the precise localization pattern of CD133 in mouse oviduct epithelial cells, we performed whole-mount three-dimensional (3D) imaging of *Fltp-H2B-mVenus* (*Flattop-driven H2B-Venus*) transgenic mice with an antibody targeted to the CD133 antigen (*n*=6 mice). *Fltp-H2B-Venus* mice contain a *LacZ-2A-H2B-Venus* cassette knocked in to the *Fltp* locus resulting in Venus expression in the nucleus of multiciliated cells (Gegg et al., 2014). In the infundibulum and ampulla of adult mice, CD133 was observed extensively at the luminal side of the epithelium with strong staining marking both individual and clusters of cells (Fig. 1A and C). In transverse sections, CD133 was observed at the apical surface of both multiciliated (white arrow) and secretory cells (yellow arrow) (Fig. 1B and D). In the ampulla-isthmus junction, CD133 was primarily co-localized with multiciliated cells (Fig. 1E and F). The distribution patterns of CD133 positive cells were generally scattered, with some coherent and continuous clusters seen in 3D reconstructions. By opening the oviduct and 3D imaging from the luminal surface, we found CD133 to be covering the apical surface of multiciliated cells and located basal to cilia (Fig. 1G). In the isthmus, CD133 was enriched on the apical surface of multiciliated cells located at the base of transverse epithelial folds (Fig. 2A,B and D). No CD133 was detected in the uterotubal junction, which also contained no multiciliated cells (Fig. 2C and E).

### CD133 becomes absent in proximal oviduct secretory cells and emerges in distal regions during multiciliated cell differentiation in neonatal oviducts

**Fig. 3.**
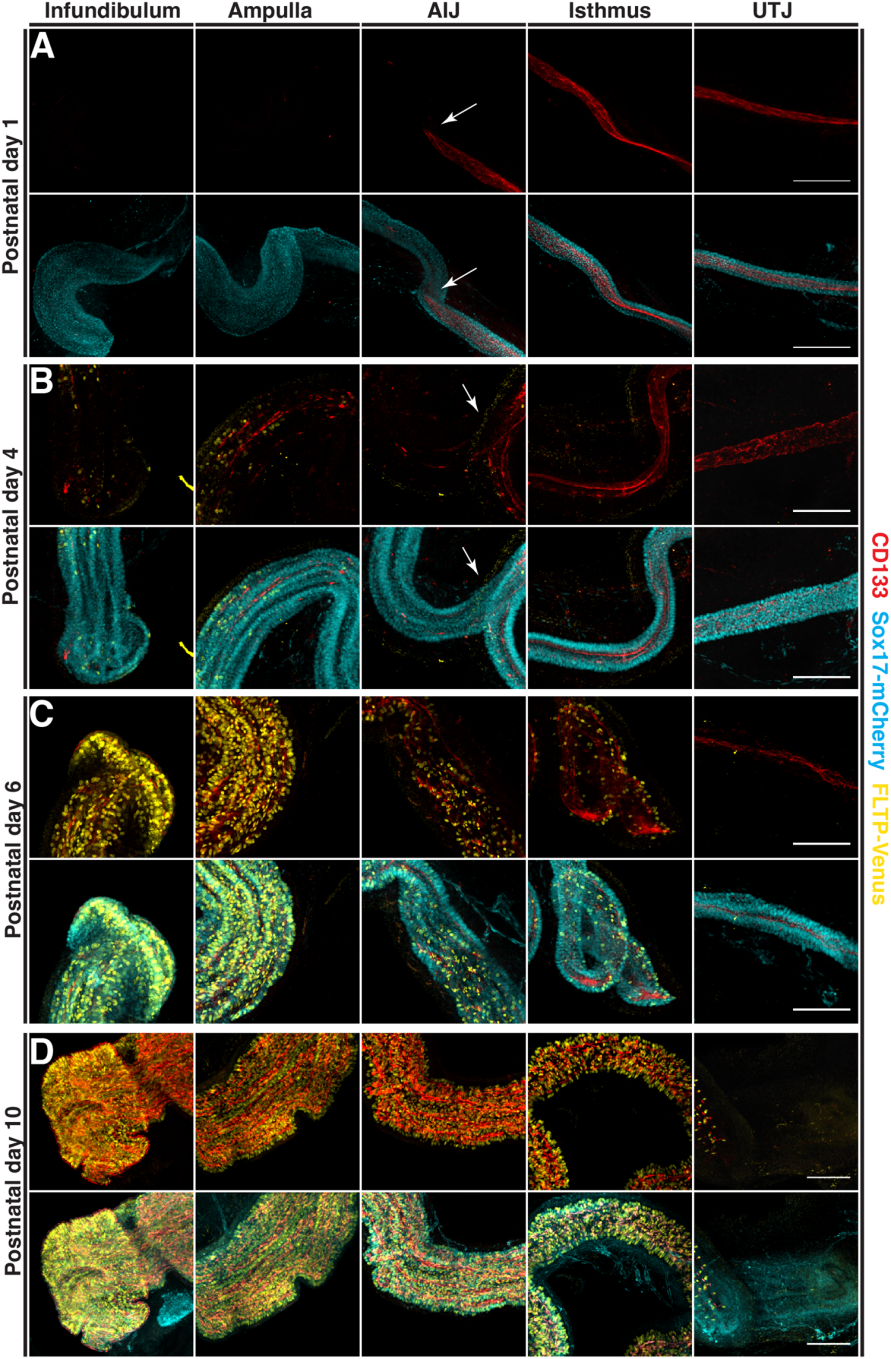
**Distribution of CD133 and multiciliated cells in the mouse oviduct during neonatal development.** The distribution patten of CD133 analyzed by immunostaining in *FLTP-H2B-Venus:Sox17-mCherry* mice. (A) Whole-mount Z projections at postnatal day 1 showing CD133 staining restricted to the isthmus and uterotubal junction with a sharp boundary at the ampulla-isthmus junction. (B) On postnatal day 4, differentiation of multiciliated cell was seen primarily in the infundibulum and ampulla and was coupled with an increase of CD133 in these regions. (C) Increased multiciliogenesis and CD133 staining was detected on postnatal day 6 in the infundibulum and ampulla. More extensive multiciliogenesis was also detected in the ampulla-isthmus junction and isthmus which was coupled with areas of higher and more discrete CD133 staining. Diffuse CD133 staining was still seen in the isthmus and uterotubal junction. (D) By postnatal day 10, CD133 in the infundibulum and ampulla resembled staining patterns seen in adult tissue. In the ampulla-isthmus junction and isthmus the pattern of CD133 and multiciliated cells had yet to fully form while CD133 was now barely detectable in the uterotubal junction. Scale bars: 100 µm.

To determine how the pattern of CD133 positive cells is established in the oviduct epithelium, we stained for CD133 in neonatal oviducts and performed 3D whole-mount imaging using *Fltp-Venus::Sox17-mCherry* mice to label multiciliated and epithelial cells respectively (Burtscher et al., 2012) (*n*=3 mice per stage). We have previously shown *Sox17* to be expressed in secretory cells of the distal oviduct and all epithelial cells in the proximal region (Harwalkar et al., 2021). At the postnatal stages analyzed, *Sox17* is initially expressed in all epithelial cells but begins to be lost in some distal epithelial cells that are undergoing multiciliogenesis (Fig. S1A). On postnatal day 1, prior to multiciliated cell differentiation, we identified uniform CD133 staining on the apical surface of epithelial progenitors in the isthmus and uterotubal junction but absent in the infundibulum and ampulla, with a sharp boundary at the ampulla-isthmus junction (white arrows in Fig. 3A and Fig. S1B), consistent with our previous observations of distinct distal and proximal epithelial populations (Harwalkar et al., 2021; Ford et al., 2021). At the onset of multiciliated cell differentiation on postnatal day 4, CD133-positive cells emerged in the infundibulum and ampulla in regions containing multiciliated cells labeled with FLTP-Venus (Fig. 3B; Fig. S1C). Cross sections of these regions confirmed CD133 present primarily on the apical surface of multiciliated cells (Fig. S1F). Similarly, to postnatal day 1, we identified a sharp boundary at the ampulla-isthmus junction with proximal regions continuing to have a more uniform pattern of CD133 staining with some areas of stronger staining and only scattered multiciliated cells (boundary marked by white arrows in Fig. 3B; Fig. S1C and F). As the proportion of multiciliated cells increased in the infundibulum and ampulla on postnatal day 6, we found a significant increase in the number of CD133-positive cells, (Fig. 3C; Fig. S1D and G). An increase in the number of multiciliated cells was also seen in the ampulla-isthmus junction and isthmus. Clear CD133-positive cells were identified within these ciliated regions with some uniform staining still present in the isthmus (Fig. 3C; Fig. S1D and G). No ciliogenesis was identified at the uterotubal junction and CD133 remained relatively uniform along the epithelium. By postnatal day 10, the adult pattern of the CD133 positive cell distribution in the infundibulum and ampulla was established, with enrichment on the apical surface of multiciliated and secretory cells (Fig. 3D; Fig. S1E and H). In the ampulla-isthmus junction and isthmus, CD133 had become absent from many epithelial cells and was predominantly associated with multiciliated cells (Fig. 3D; Fig. S1E and H). CD133 staining was absent from the uterotubal junction by this stage (Fig. 3D; Fig. S1H). Taken together, our observations identified a dynamic expression of CD133 during neonatal development in the oviduct epithelium. CD133 initially absent from distal regions, started to mark epithelial cells during multiciliated cell differentiation while the CD133 positive cells in the proximal oviduct become negative in non-multiciliated cells.

### *Prom1* expression is in agreement with CD133 staining in *Prom1^C-L:C-L^:Rosa26^Tdtomato:Tdtomato^* mice

**Fig. 4.**
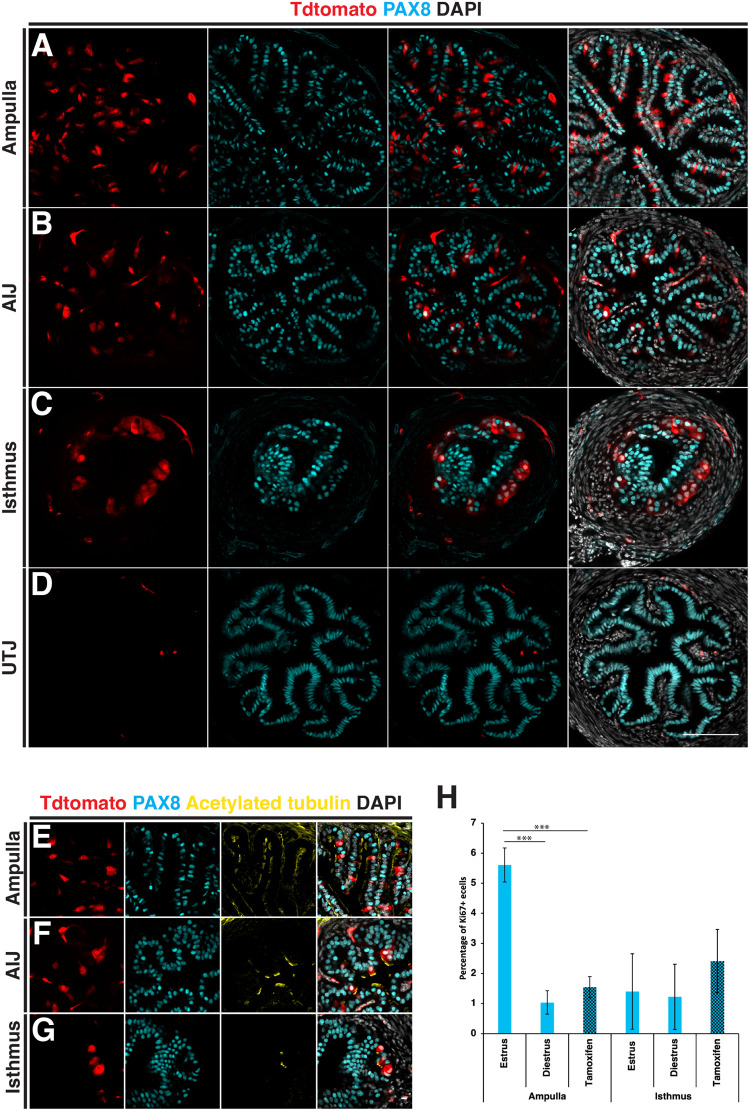
**Distribution of *Prom1*-expressing cells in the adult mouse oviduct.** The distribution pattern of *Prom1* expressing cells was analysed in *Prom1^C-L:C-L^:Rosa26^Tdtomato:Tdtomato^* mice, by tamoxifen injection on 5 consecutive days to label *Prom1*-expressing cells and dissection of oviducts after 72 h. (A) Transverse section of the ampulla showing a diffuse pattern of Tdtomato-labeled cells counter stained with a PAX8 antibody to label secretory cells. (B) Fewer Tdtomato-labeled cells were detected in the ampulla–isthmus junction. (C) Tdtomato-labeled cells were restricted to the base of epithelial folds in the isthmus. (D) No Tdtomato-labeled cells were identified in the epithelial cells at the uterotubal junction. (E-G) Counter staining with cilia marker acetylated tubulin, revealed the majority of Tdtomato cells to be multiciliated in the ampulla, ampulla–isthmus junction and isthmus. (H) A comparison of the proportion of Ki67- positive epithelial cells during the oestrus cycle and 6 h after tamoxifen administration. No significant changes were detected in the isthmus, while significantly more Ki67-positive cells were detected during estrus compared to diestrus and after tamoxifen administration. Statistical tests used in H=two-tailed Student's *t*-test. Scale bars: A-D, 100 µm; E-G, 10 µm.

In order to investigate the fate and generative potential of *Prom1* expressing cells, we used a lineage tracing approach with homozygous *Prom1^C-L:C-L^:Rosa26^Tdtomato:Tdtomato^* mice (Zhu et al., 2009; Madisen et al., 2010). In these mice *Prom1* expression is absent due to the insertion of a *CreERT2-nLacZ* transgene into the *Prom1* locus. To determine if the expression from the *Prom1* locus was consistent with the CD133 expression pattern, tamoxifen was administered on 5 consecutive days followed by a 72-h lag. Oviducts were then dissected and sectioned using a vibratome. Due to technical difficulties in obtaining consistent sections of the infundibulum by vibratome sectioning, our analysis of the distal oviduct was restricted to the ampulla. Using PAX8 and acetylated tubulin to label secretory and multiciliated cells respectively, we found the pattern of Tdtomato labeled cells closely mirrored the staining seen with the CD133 antibody (Fig. 4A-D). In the ampulla, Tdtomato primarily labeled multiciliated cells and was sporadically distributed throughout the epithelium (Fig. 4A and E). The ampulla-isthmus junction which contained significantly fewer multiciliated cells had fewer Tdtomato-positive cells which were primarily multiciliated (Fig. 4B and F). In the isthmus, Tdtomato-positive cells were restricted primarily to multiciliated populations at the base of epithelial folds, which we previously showed to have high CD133 antibody staining (Fig. 4C and G). No labeled cells were detected in the uterotubal junction (Fig. 4D), and in all regions Tdtomato did not show colocalization with proliferation marker Ki67 (0 Ki67+/Tdtomato+ cells, *n*=3 mice, (289 Ki67+ cells) (Fig. S2A).

Tamoxifen is an estrogen receptor modulator and has been reported to induce hyperplasia in the mouse oviduct epithelium after acute prenatal and sustained adult exposure (Diwan et al., 1997; Niwa et al., 1998). To determine the effects of an acute adult exposure of tamoxifen, we administered five doses of tamoxifen and collected oviducts 6 h after the final dose. Compared to homeostatic levels of proliferation, measured by Ki67 staining, we identified no significant difference in proliferation in the isthmus after tamoxifen exposure, and the proportion of Ki67-positive cells fell within the normal range seen during the estrous cycle in the ampulla (Fig. 4H and Fig. S2B) [*n*=3 mice per condition/stage (*P=*>0.05, two-tailed students *t*-test)]. Although this result suggests an acute dose of tamoxifen in adult mice is not having a significant impact on homeostasis, the possible side effects of tamoxifen in this system should be considered when evaluating the results presented.

### *Prom1* expressing cells have a low generative capacity and progressively migrate to the ridge of epithelial folds in the ampulla

**Fig. 5.**
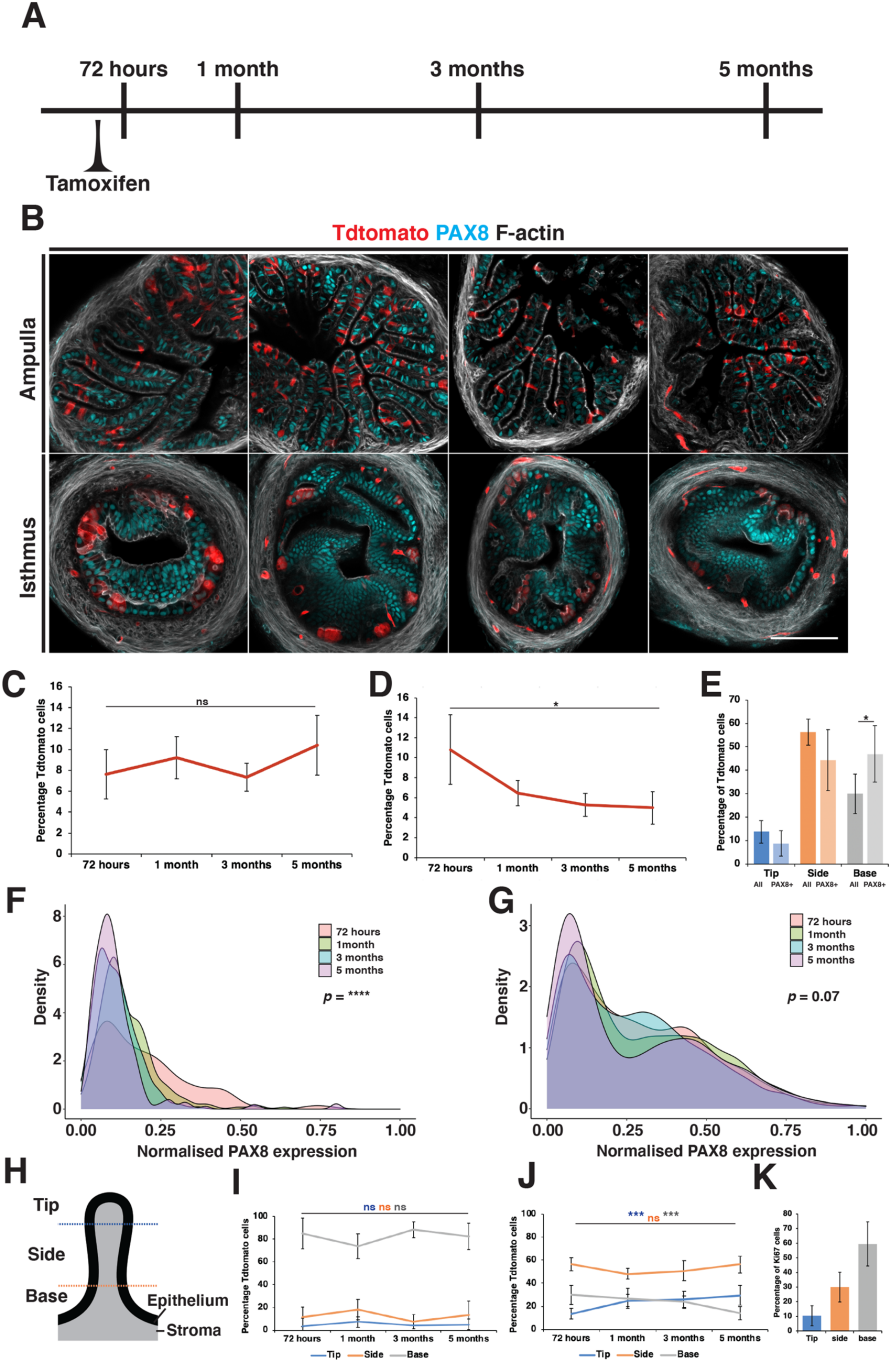
**Lineage tracing *Prom1*-expressing cells in the mouse oviduct**. (A) Lineage tracing was performed in *Prom1^C-L:C-L^:Rosa26^Tdtomato:Tdtomato^* mice, by administration of five doses of tamoxifen on consecutive days followed by collection of oviduct at different time points over a 5-month period. (B) Selected examples showing the distribution pattern of labeled cells over 5 months in the ampulla and isthmus. Panels show time points from left to right after 72 h, 1 month, 3 months and 5 months. (C) No significant change in the proportion of Tdtomato-positive cells was detected in the ampulla. (D) In the isthmus, a steady drop in the proportion of Tdtomato-positive cells was seen. (E) In the ampulla, Tdtomato-positive cells were found in all regions of epithelial folds. Tdtomato-positive cells that also had visible PAX8 staining were also found in all regions but were overrepresented at the base of epithelial folds. (F) Density plot showing the distribution of PAX8 expression in Tdtomato-positive cells over 5 months of lineage tracing. Low PAX8 expression was detected in a subpopulation of cells at 72 h. After 1 month almost all Tdtomato cells had no detectable levels of PAX8 expression (*n*=238 cells per time point). (G) Density plot showing the distribution of PAX8 expression of all epithelial cells from the same lineage experiment (*n*=2438 cells per time point). (H) Schematic of an oviduct epithelial fold in a transverse section showing the segmentation method used to classify epithelial cell position. (I) Line graph showing the proportion of Tdtomato-positive cells within each region throughout the lineage tracing in the isthmus, showing no significant changes. (J) In the ampulla we detected a significant increase in the proportion of Tdtomato-positive cells at the ridge and a decrease at the base over 5 months. (K) Quantification of the proportion of Ki67-positive cells by location, showing the majority to be located at the base of epithelial folds (*n*=5 mice, 226 cells). Scale bar in B: 100 µm. Statistical tests used in C-E, I and J=two-tailed Student’s *t*-test, F and G=two-sample Kolmogorov–Smirnov test comparing 72-h and 1-month distributions. *N*=3 mice for all lineage tracing data.

To determine the fate of *Prom1*-expressing cells, five injections of tamoxifen were administered over consecutive days and oviducts collected over a 5-month period (Fig. 5A). We detected no significant change in the proportion of Tdtomato-positive cells in the ampulla and a slight decrease in labeled cells in the isthmus (*n*=3 mice per time point) (Fig. 5B-D). To follow the differentiation of *Prom1*-expressing cells over time we calculated the intensity of PAX8 expression in Tdtomato labeled cells. In the ampulla 20% of cells had a visually detectable level of PAX8 staining after 72 h and were distributed in all regions of epithelial folds but proportionally enriched at the base (Fig. 5E). Of these PAX8-positive cells the majority had a relativity low level of PAX8 staining compared to the total epithelial cell population (Fig. 5F and G). Over the course of the lineage tracing experiment, we detected a rapid decrease in the proportion of PAX8-expressing Tdtomato-postive cells to 1% after 1 month which was sustained (Fig. 5F). In the isthmus, however, we did not observe this due to the ubiquitous expression of PAX8 in all epithelial cells.

To determine the movement of *Prom1*-expressing cells, each labeled cell during lineage tracing was assigned a position in relation to the base, side and tip of epithelial folds in transverse sections (Fig. 5H). In the isthmus labeled cells remained restricted to clusters of cells at the base of epithelial folds (Fig. 5I). In the ampulla however, we detected a progressive increase in the proportion of labeled cells at the ridge of epithelial folds and a decrease at the base (Fig. 5J), suggesting a base to ridge drift of epithelial cells over time. In keeping with this observation, the majority of proliferating cells (Ki67+) in the ampulla were detected at the base of epithelial folds (Fig. 5K).

## DISCUSSION

CD133/*Prom1* is a well characterized adult and cancer stem cell marker in many tissues (Yin et al., 1997; Miraglia et al., 1997; Uchida et al., 2000; Sagrinati et al., 2006; Richardson et al., 2004; Glumac and Lebeau, 2018). In the present study, we investigated the expression pattern and generative capacity of CD133/*Prom1*-expressing cells in the mouse oviduct epithelium. We determined that CD133 labeled proximal oviduct epithelium progenitors prior to multiciliated cell differentiation but was absent from the infundibulum and ampulla. CD133-positive cells emerged in distal regions during multiciliated cell differentiation and became restricted to multiciliated cells in the isthmus and ampulla-isthmus junction. *Prom1*-expressing cells that were analyzed using *Prom1^C-L:C-L^:Rosa26^Tdtomato:Tdtomato^* mice, showed a similar distribution pattern in adult mice. Using lineage tracing we showed that cells that had expressed *Prom1* did not expand over time and in the case of the isthmus decreased over the course of the experiment. As the labeling of *Prom1* expressing cells was not statured, the stable number of labeled cells could be attributed to neutral competition between labeled and unlabeled *Prom1*-expressing cells. However, we found no evidence of clonal expansion and near complete differentiation of labeled cells after 1 month. Taken together, our results suggest that CD133/*Prom1* does not label a stem or progenitor population in the adult mouse oviduct.

Multiciliated cell half-life in the distal mouse oviduct has been estimated to be around 6 months (Roberson et al., 2020). As we see a near exclusive labeling of multiciliated cells one month after tamoxifen injections we would have expected to see a steady drop in the number of labeled cells over time. However, in the ampulla we identified no significant change in the proportion of labeled cells over 5 months. We also found that *Prom1* labels a population of cells in the ampulla with low PAX8 expression that rapidly transition into multiciliated cells. Considering the slow turnover of *Prom1* expressing cells it is possible that *Prom1* may mark PAX8-positive cells differentiating into multiciliated cells but is subsequently lost from aged multiciliated cells. The differences between the ampulla and isthmus may reflect a broader expression of *Prom1* in the isthmus multiciliated population or a different turnover rate.

CD133 has recently been identified on the apical surface of lung epithelial progenitors and becomes restricted to multiciliated cells during differentiation (Serra et al., 2022). *Prom1* expression was shown to be regulated by Notch activity in multiciliated cells, which regulated cilia length and impacted ciliary function. CD133 levels in the lung are in a gradient, with higher staining in the bronchiole compared to the bronchus, suggested cilia function is regulated along the length of the airways by PROM1 activity. It is possible that PROM1 has a similar role in regulating cilia function in the oviduct. In a previous publication we identified *Prom1* and Notch target gene *Hes1* to be significantly over expressed in multiciliated cells of the isthmus compared to the infundibulum and ampulla (Ford et al., 2021). Another report has also detailed dynamic expression of Notch signaling components along the length of the oviduct during the oestrous cycle (Murta et al., 2016). These findings could indicate a potential mechanism for regulating multiciliated cell function in different regions of the oviduct and during different stages of the oestrous cycle. Although, in homozygous *Prom1^C-L:C-L^:Rosa26^Tdtomato:Tdtomato^* mice which had lost *Prom1* expression we detected no impact on female fertility, indicating that any role PROM1 may have in regulating cilia length or function is not required for successful fertilization or preimplantation development.

The oviduct is morphologically segmented into four distinct regions containing varying proportions of multiciliated and secretory cells (Stewart and Behringer, 2012). The homeostasis of the entire oviduct epithelium is generally considered as a single population. Several reports have however started to highlight differences between the distal and proximal epithelial cell populations, showing a concentration of label retaining cells, cells expressing known adult stem cell markers and organoid forming cells in the distal region of the oviduct (Alwosaibai et al., 2017; Ng et al., 2013; Paik et al., 2012; Patterson and Pru, 2013; Wang et al., 2012; Xie et al., 2018). Two other reports from our lab have further highlighted these differences, showing distinct expression patterns between epithelial cells in these two regions and identified the formation of distal-proximal lineages early in Müllerian duct development (Ford et al., 2021; Harwalkar et al., 2021). In our lineage-tracing study, we find that the distribution pattern of *Prom1*-expressing cells and the drift of these cells is distinct between the ampulla and isthmus. In addition, only epithelial cells in the ampulla responded to changes during the oestrus cycle resulting in an increase in proliferation during estrus. These results support the notion of independent homeostatic mechanisms within the morphologically distinct regions of the oviduct.

## MATERIALS AND METHODS

### Mouse stains and injections

All animal work was approved by the internal ethics committee at McGill University and undertaken at the Goodman Cancer Institute animal facility. *Prom1C-L* (#017743) and *Tdtomato*^flox/flox^ mice (Ai14, #007914) were acquired from JAX and maintained on a C57BL/6 background. *Fltp-H2B-Venus* (Gegg et al., 2014) and *Sox17-mCherry* (Burtscher et al., 2012) mice were generated and received as a kind gift from Dr Heiko Lickert and Dr Ingo Burtscher (IDR Munich, Germany). C57BL/6 stock mice were used as wild-type mice. All experiments unless otherwise stated, were performed on at least 2-month-old, sexually mature females. Tamoxifen was prepared on the day of injection at a concentration of 20 mg/ml in corn oil and 4 mg per 25 g administered by intraperitoneal injection.

### Immunofluorescence

Neonatal and adult oviducts were dissected in PBS and fixed in 4% w/v PFA/PBS (Polysciences) for 30 min at room temperature followed by 3 PBS washes. Ducts were cryoprotected through a sucrose/PBS gradient 4°C, allowing time for the ducts to sink to the bottom of the tube between each gradient. Ducts were embedded in OCT mounting solution (Fisher HealthCare) and snap frozen on dry ice before being stored in a −80°C freezer. 10 µm sections were cut using the microtome cryostat Microm HM525 (ThermoFisher Scientific) mounted on Superfrost glass slides, air dried and stored in −80°C freezer. For immunofluorescence, sections were allowed to thaw for 10 min at room temperature followed by rehydration with PBS. Sections were permeabilized for 5 min with 0.5% v/v Triton-X/PBS (Sigma) and then blocked with 10% v/v Fetal Bovine Serum (FBS) (Wisen Bioproducts), 0.1% v/v Triton-X in PBS for 1 h at room temperature. Primary antibodies were diluted in 1% v/v FBS, 0.1% v/v Triton-X in PBS to working concentrations (1/250) and incubated overnight in a dark humidified chambered at 4°C. The primary antibodies used in this study were: PAX8 (proteintech #10336-1-AP), acetylated tubulin (Sigma-Aldrich #T7451), CD133 (ThermoFisher Scientific, monoclonal 13A4 #14-1331-80), Ki67 (ThermoFisher Scientific #14-5698-82), anti-GFP (Abcam, #ab13970) and anti-mCherry (Abcam, #ab213511). The following day, sections were washed before incubation with secondary antibodies (ThermoFisher Scientific, Alexa Fluor, diluted 1/400 in 1% v/v FBS, 0.1% v/v Triton-X in PBS) and for 1 h in a dark humidified chamber at room temperature. In some cases, Phalloidin (ThermoFisher Scientific, 1/500) was added to label f-actin. Sections were then washed again with 5 µg/ml DAPI (ThermoFisher Scientific) added to the final wash step before sections were mounted with prolong gold (Invitrogen) for imaging. For lineage tracing and adult CD133 transverse sections, fixed samples were embedded in 4% agarose and sectioned using a vibratome into 100 µm sections. The sections were then permeabilized for 10 min in 0.5% v/v Triton-X/PBS, stained as above and then mounted in prolong gold between two coverslips separated by a spacer (Invitrogen Sercure-seal 0.12 mm).

### Whole-mount immunostaining, tissue clearing, and 3D confocal imaging

Mouse female reproductive tracts were collected and straightened by removing the mesosalpinx. The straightened oviduct was fixed with DMSO (Dimethyl sulfoxide, Sigma-Aldrich D8418): methanol in the ratio 1:4 and cut into three to four pieces prior to placing at −20°C overnight. For the surface view of multiciliated cells, a single longitudinal cut was made along the length of the oviduct and the oviduct opened to expose the luminal surface which was then fixed as described above. The antibody staining protocol has been previously described in (Arora et al., 2016). Blocking was done overnight in solution containing 1% Triton X-100 (Sigma-Aldrich, T8787), 2% powdered milk, and 1× phosphate-buffered saline (PBMST; PBS, BioShop Canada Inc., PBS405). Primary and secondary antibody staining were performed in PBSMT for 5 and 2 days, respectively, at 4° on a shaker. After six 30min long PBSMT washes, the oviduct pieces were transferred successively to methanol:PBT (1:1, PBT: 1× PBS and 1% Triton X-100), 100% methanol (BioShop Canada Inc., MET302.1), 3% H2O2 (BioShop Canada Inc., HYP001.1), and 100% methanol prior to benzyl alcohol/benzyl benzoate (BABB) clearing. BABB-cleared samples were placed on a #1.5 coverslip (ThermoFisher Scientific, 12-545F) with 10–15μl BABB prior to imaging using the 10× objective (numerical aperture 0.30).

### Imaging and statistics

All imaging was performed on an LSM 800 confocal microscope (Zeiss) at the advanced bioimaging facility (McGill University). Image analysis was conducted with FIJI using custom designed software (Schindelin et al., 2012). Statistical analysis was then performed either using Excel (Microsoft) or R-studio.

## Supplementary Material

10.1242/biolopen.059963_sup1 Supplementary information
