# Supplementary figures and images for: CD133/Prom1 marks proximal mouse oviduct epithelial progenitors and adult epithelial cells with a low generative capacity
Source: Biol Open. 2023 Sep 13;12(9):bio059963. doi: 10.1242/bio.059963 (PMC10508696; doi:10.1242/bio.059963)

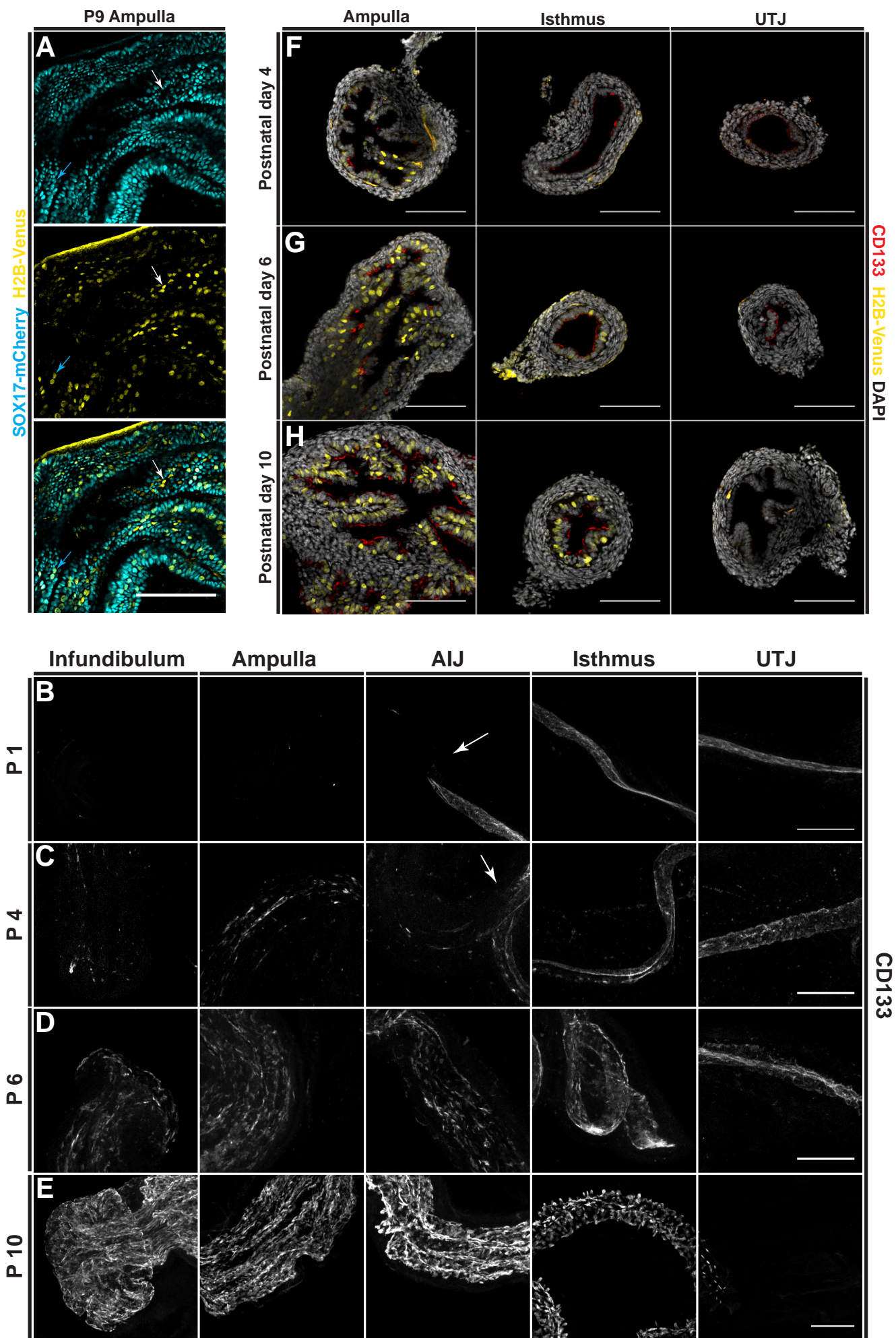

Fig. S1.

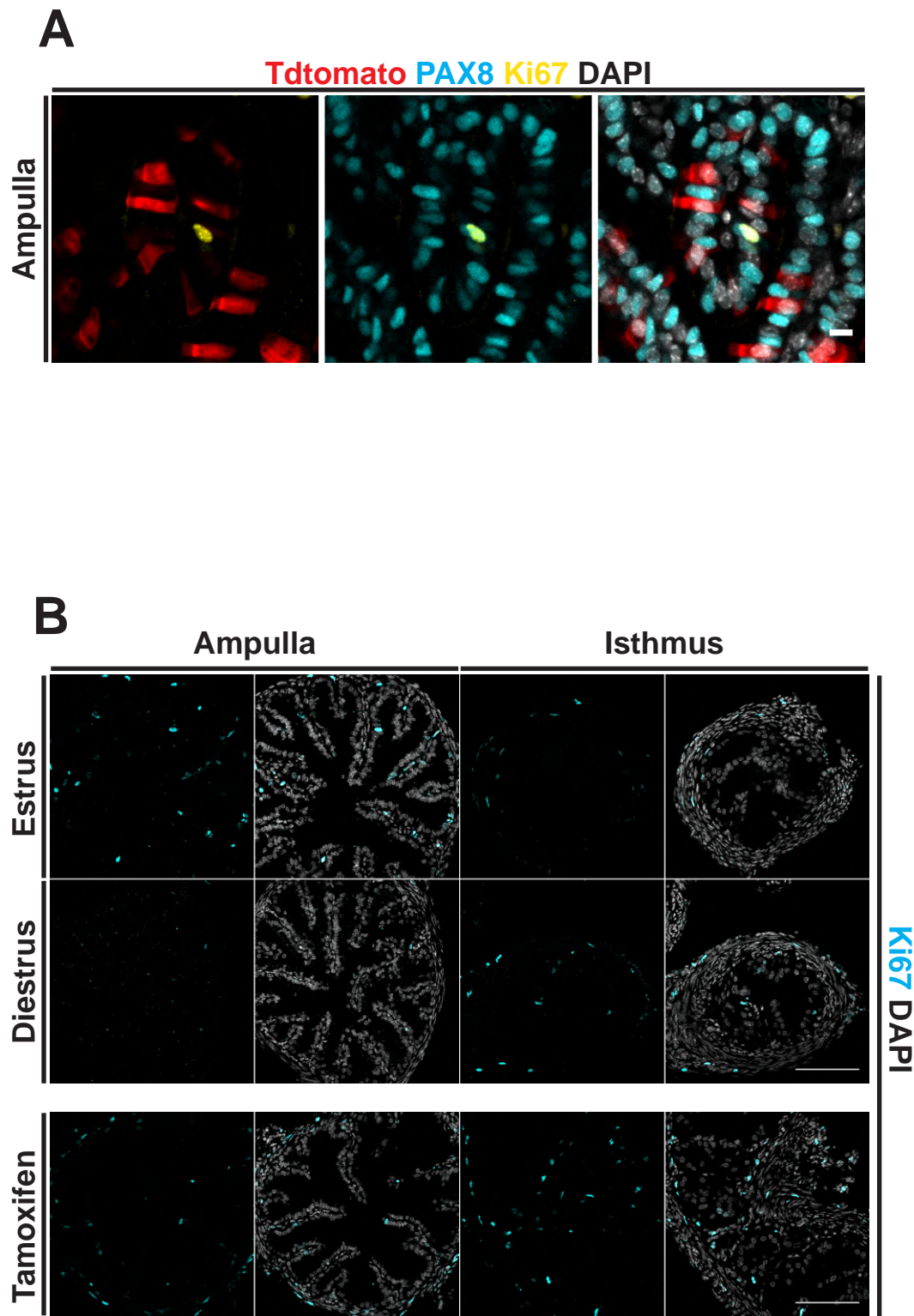

Fig. S2.

Supplement: Supplementary information [file biolopen-12-059963-s1.pdf]
